# Anticardiolipin Antibodies Presenting With Acute Renal Infarction in a Healthy 26-Year-Old Female

**DOI:** 10.7759/cureus.26088

**Published:** 2022-06-19

**Authors:** Caitlyn Cross, James Cappola

**Affiliations:** 1 Internal Medicine, Campbell University School of Osteopathic Medicine, Lillington, USA

**Keywords:** thrombophilia, hyper coagulable state, anti-cardiolipin antibodies, antiphospholipid syndrome, oral contraceptives, renal infarction

## Abstract

A 26-year-old female presented to the emergency department with right lower quadrant pain. This pain lasted a couple of days, worsened, and was associated with nausea but no vomiting. Upon presentation, physical examination showed tenderness to palpation in the right lower quadrant with bilateral purple non-blanching discolorations present on her toes and no costovertebral angle tenderness. Contrast-enhanced computed tomography (CT) showed acute right renal infarction. Thrombophilia workup was done, which showed elevated antinuclear antibodies and anticardiolipin antibodies. Elevated lactate dehydrogenase, c-reactive protein, and sedimentation rate were also found. No other inherited thrombophilia was discovered in lab work. Combined oral contraceptives were stopped, and the patient was started on enoxaparin followed by rivaroxaban upon discharge.

The antiphospholipid syndrome commonly presents in young females as recurrent miscarriages, stroke, or deep venous thrombosis. Venous thrombosis is more common than arterial. Rare arterial thrombosis manifestations of this syndrome include coronary, retinal, mesenteric, and renal. This is a rare case of anticardiolipin antibodies presenting as an acute right renal infarction. This raises the question if clinicians should screen for inherited thrombophilia before prescribing oral contraceptives.

## Introduction

Anticardiolipin antibodies are found in antiphospholipid syndrome (APS). APS is multifactorial but thought to be due to an autoimmune process. It can present as primary APS (without lupus) or secondary APS (with lupus). This syndrome can present in many ways. It can present with venous or arterial thromboembolism (examples of these common presentations include deep venous thrombosis or stroke) [1]. In this example, however, it presented with acute renal infarction in a healthy 26-year-old female. Acute renal infarctions are rare with an incidence rate of about 0.004%-0.007% [2] and normally present in adults with a history of atrial fibrillation [3]. We present a rare case of acute renal infarction in the setting of anticardiolipin antibodies.

This article was previously presented as a poster presentation at the 2021 North Carolina Society of the American College of Osteopathic Family Physicians on August 14, 2021.

## Case presentation

A 26-year-old female presented to the emergency department with right lower quadrant pain that awoke her from sleep. Her pain continued to worsen over several days to the point that any movement, especially movement of the right leg, made it unbearable. Her only associated symptoms included lack of appetite and nausea but no vomiting. The patient has no past medical history, pregnancies, or miscarriages, is healthy, and runs 5-10 miles three times a week. Her only medication is a combined birth control pill (levonorgestrel and ethinyl estradiol 0.15 mg/0.03 mg) which she was prescribed at age 13 due to menorrhagia. She is a nonsmoker. The patient denied any family history of inherited thrombophilia, lupus, or other autoimmune conditions. However, both of her sisters have congenital heart defects including patent foramen ovale (PFO), patent ductus arteriosus, aortic coarctation, aortic regurgitation, and mitral regurgitation.

**Figure 1:**
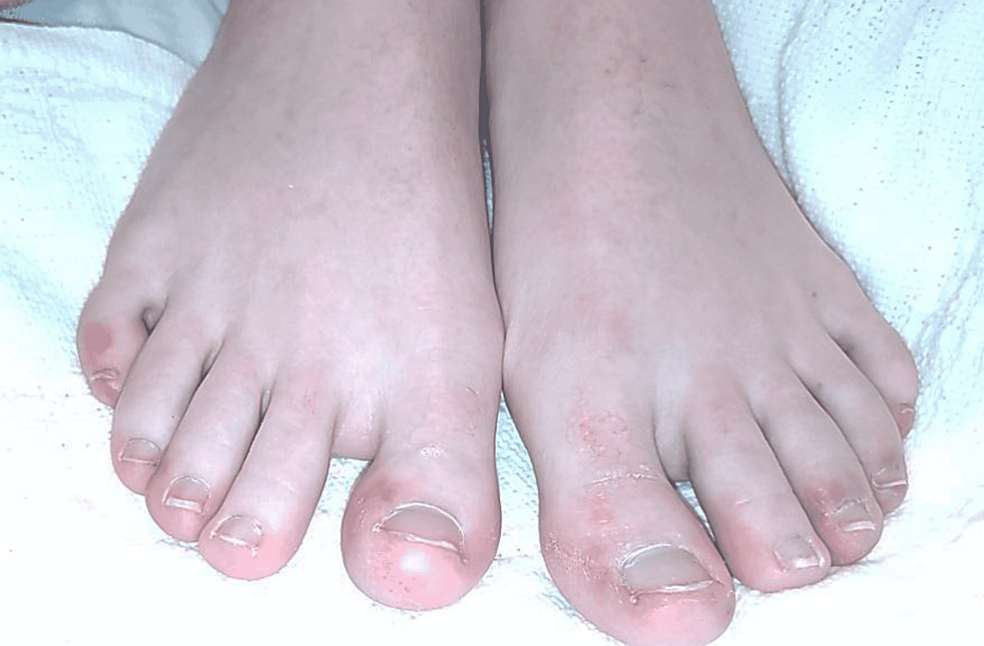
Bilateral purple non-blanching lesions on the patient’s toes.

Her vital signs on presentation included blood pressure 124/84, heart rate 92, respirations 16, oxygen saturation of 100% on room air, and body mass index (BMI) of 18.3. Physical examination showed tenderness to palpation in the right lower quadrant with no costovertebral angle tenderness. Bilateral purple non-blanching discolorations were present on her toes consistent with livedo reticularis. The patient stated that her condition of the toes worsened over the last six months (Figure 1).

**Figure 2:**
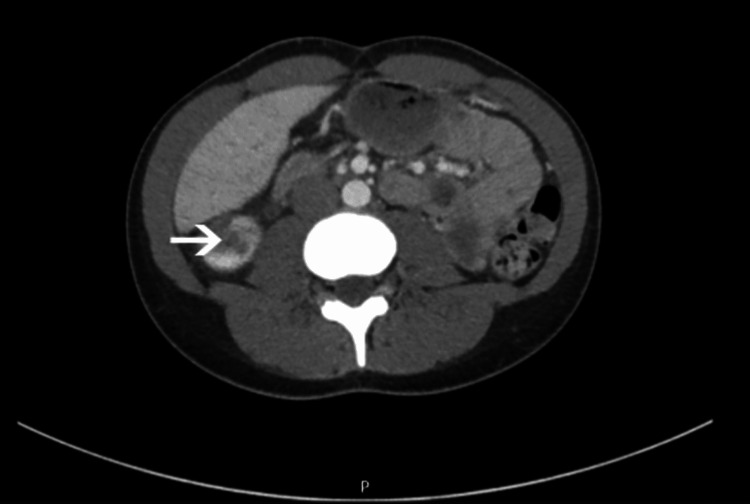
Contrast-enhanced abdominal computed tomography showing a hypodense region in the central right kidney (white arrow).

**Figure 3:**
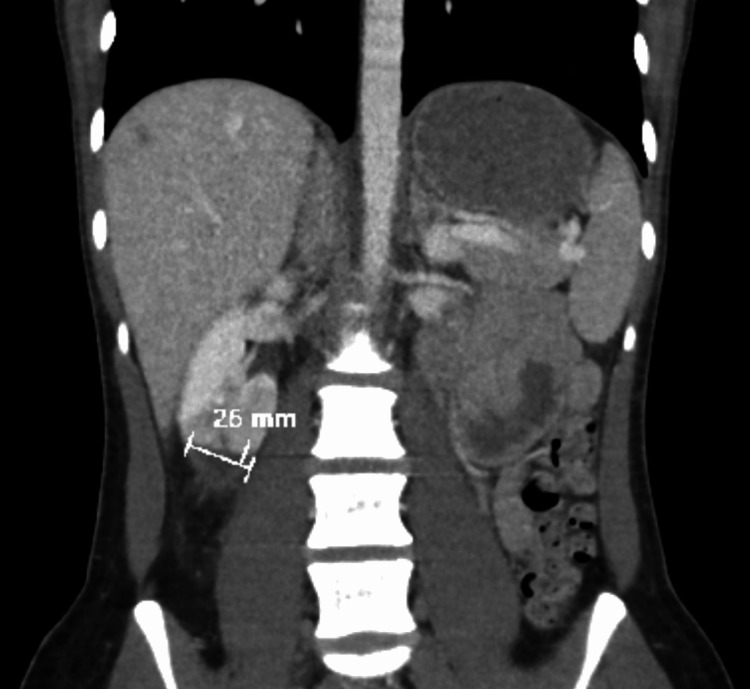
Coronal view of the contrast-enhanced abdominal computed tomography showing 26 mm right kidney infarction

A computed tomography (CT) scan with contrast was ordered that showed acute right renal infarction (Figures 2, 3). The patient was then admitted for further workup. Electrocardiogram (EKG) and cardiac telemetry showed normal sinus rhythm with no signs of atrial fibrillation. A transthoracic echocardiogram showed no abnormalities. Urinalysis showed hematuria and eosinophils. Complete blood count showed leukocytopenia at 3.2 x 10^3 ^WBC/mm^3^. CT angiography with aortic runoff showed infarction of the right kidney inferior pole. The patient was normotensive with normal kidney function, blood urea nitrogen 4 mg/dL, and creatinine 0.8 mg/dL.

Further workup was done including inherited thrombophilia and connective tissue disorders. Tests for Factor V Leiden, antithrombin, protein S, protein C, and homocysteine were all normal. The rest of the labs are shown in Table 1.

**Table 1:** Autoimmune and connective tissue disorders lab results

Test	Result	Reference Range
Anti-nuclear antibody (ANA)	1:1280, Speckled pattern	Negative
Anti-Sjögren's-syndrome-related antigen A autoantibodies (anti-SSA)	7.3 AI	0.0-0.9 AI
Anti-Sjögren's-syndrome-related antigen B autoantibodies (anti-SSB)	>8.0 AI	0.0-0.9 AI
Cardiolipin antibody IgM	19 MPL U/mL	0-12 MPL U/mL
Lactate dehydrogenase (LDH)	277 U/L	110-270 U/L
C-reactive protein (CRP)	49.01 mg/L	≤5.00 mg/L
Sedimentation rate	31 mm/h	0-20 mm/h

The patient was given enoxaparin 50 mg subcutaneously twice daily. She was also given normal saline at 125 mL/h. On hospital day 2, her right lower quadrant pain had mostly resolved. She was then transitioned to oral rivaroxaban starting at 15 mg twice daily for 21 days then 20 mg daily. Her oral contraceptive pill was stopped, and she was told to follow up closely with her primary care physician.

## Discussion

Acute renal infarction is a rare cause of low flank pain. Other more common things on the differential include nephrolithiasis, pyelonephritis, appendicitis, or ovarian torsion [4]. Lack of pyuria, leukopenia, and findings on imaging allowed pyelonephritis to be ruled out in this case. Similarly, lack of hematuria or a stone seen on CT ruled out nephrolithiasis. Appendicitis was also ruled out due to a normal-appearing appendix on contrast-enhanced CT. Ovarian torsion was also ruled out due to normal-appearing ovaries.

The most frequent cause of acute renal infarction is thromboembolism due to atrial fibrillation [3]. Our patient was not in atrial fibrillation and there was low suspicion of atrial fibrillation due to the age and health of the patient. Other causes of thromboembolism include a PFO or atrial septal defect, particularly given the age of the patient and family history of congenital heart defects. However, transthoracic echo showed no abnormalities. Another cause of acute renal infarction is a hypercoagulable state, most commonly Factor V Leiden. Other hypercoagulable states include protein C and S deficiency, hyperhomocysteinemia, antithrombin III deficiency, medication side effect, and antiphospholipid antibodies [4]. In this patient’s case, her antiphospholipid antibodies came back positive; in particular, her anticardiolipin IgM antibody.

APS is a hypercoagulable state that occurs with or without lupus. To meet the criteria for diagnosis, one antibody must be positive (lupus anticoagulant, anticardiolipin, or anti-beta-2 glycoprotein) and a patient must have either a thrombotic event or a pregnancy complication. APS presents more commonly in women between the ages of 20 and 50. Common presentations of this condition include recurrent pregnancy losses, stroke, or transient ischemic attack. Other common places in which the thromboembolic events occur in people with APS include the coronary, retinal, mesenteric, and, as in this case, renal arteries [1]. Renal infarction tends to be the rarest event in APS [3].

The patient’s use of combined oral contraceptives likely contributed to her acute renal infarction. Combined oral contraceptives are associated with an increased risk of venous thromboembolism with the highest risk in women over the age of 35 who smoke [5]. Arterial thromboembolism in the setting of combined oral contraceptives is rarer than venous thromboembolism but it has been reported [6]. Our patient’s presentation raises the question if clinicians should screen women for a hypercoagulable state before starting a combined oral contraceptive. Currently, the guidelines state that if the patient is younger than 35 years of age, nonsmoker, with no previous thrombotic events, migraines, or autoimmune conditions, combined oral contraceptives are a safe treatment for menorrhagia with no increased risk of adverse events compared to taking naproxen and danazol [7]. However, to prevent future cases, should a thrombophilia and autoimmune workup be done before prescribing combined oral contraceptives. This case raises that question.

## Conclusions

Renal infarction is rare and even more so as the first presentation of APS. This patient was also taking combined oral contraceptives which also has a known association with arterial and venous thromboembolism. This patient’s diagnosis raises the question if clinicians should screen for a hypercoagulable state before putting young, otherwise healthy women on contraceptives. Our patient also demonstrates that the differential for acute renal infarction includes underlying APS, especially in a young healthy patient.
